# CXorf67 in malignancies: Deciphering epigenetic landscapes and clinical implications for precision oncology

**DOI:** 10.1016/j.gendis.2025.101933

**Published:** 2025-11-10

**Authors:** Xinning Yu, Huatao Wu, Yangzheng Lan, Wenjia Chen, Jing Liu

**Affiliations:** a The Breast Center, Cancer Hospital of Shantou University Medical College, Shantou, Guangdong 515041, China; b Department of General Surgery, The First Affiliated Hospital of Shantou University Medical College, Shantou, Guangdong 515041, China; c Department of Physiology, Shantou University Medical College, Shantou, Guangdong 515041, China

**Keywords:** CXorf67, DNA damage, Epigenetics, Malignancies, Therapeutic target

## Abstract

The emerging focus on epigenetic regulation in cancer biology has unveiled the significant role of CXorf67, a protein encoded by a gene on the X chromosome. CXorf67 interacts with core components of PRC2, namely EZH2 and SUZ12, thereby influencing histone modifications like H3K27me3. Research indicates that CXorf67 is overexpressed in specific malignancies, including posterior fossa ependymomas, diffuse midline glioma, endometrial stromal sarcoma, non-small cell lung cancer, and Merkel cell carcinoma. In posterior fossa ependymomas and diffuse midline glioma, CXorf67 mimics the oncogenic histone H3K27M, inhibiting PRC2 function and altering chromatin states. In endometrial stromal sarcoma, CXorf67 forms fusion genes with MBTD1, potentially disrupting polycomb group (PcG) functions. Additionally, CXorf67's interaction with PALB2 affects the BRCA1-PALB2-BRCA2 complex, influencing DNA repair mechanisms. These findings highlight CXorf67's dual role in epigenetic regulation and DNA damage response, suggesting its potential as a therapeutic target. However, further research is needed to explore its functions in other cancers and clarify its molecular mechanisms. Our review synthesizes current knowledge on CXorf67's biological significance, particularly in epigenetics and DNA damage, and its implications in oncogenesis.

## Introduction

The Human Genome Project (HGP), launched in 1990 and completed in 2003, represents one of the most ambitious and transformative endeavors in modern biology. As a large-scale international collaboration, the HGP successfully sequenced the entire human genome, comprising approximately 3 billion base pairs, thereby providing an unprecedented reference map of human genetic information. This foundational achievement not only enabled a deeper understanding of gene structure and function but also revolutionized biomedical research by accelerating the identification of disease-associated genes and facilitating the development of targeted therapies and personalized medicine.^1^, ^2^, ^3^ Notably, the HGP also uncovered a large number of open reading frames (ORFs) encoding hypothetical proteins, whose biological functions remain largely unknown. Many of these genes are expressed in specific tissues or developmental stages, suggesting potential roles in human growth, development, and disease. Chromosome X open reading frame 67 (CXorf67), located on chromosome Xp11.22, is one such gene. It encodes a protein of approximately 503 amino acids with no well-defined domains, and has recently gained attention due to its aberrant expression in certain malignancies.^4^ However, its physiological function and regulatory mechanisms in normal tissues remain elusive, warranting further investigation.

Epigenetic mechanisms regulate gene expression without altering the underlying DNA sequence, primarily through modifications of DNA and histone proteins. Histones, particularly H3, H2A, H2B, and H4, assemble into nucleosomes, the fundamental units of chromatin. These nucleosomes wrap around DNA and play a pivotal role in genome organization, thereby influencing gene accessibility and transcriptional activity.^5^ Over the past decade, the covalent modification of chromatin and its structural organization have emerged as central regulators of gene expression, and their relevance to cancer has become a major focus in the field of cancer biology.^6^ Among these modifications, H3K27me3 is a key repressive epigenetic mark, essential for gene silencing and the maintenance of higher-order chromatin structure. The polycomb repressive complex 2 (PRC2) is the principal enzymatic complex responsible for the establishment and maintenance of H3K27 trimethylation. Since its identification in 2019 as interacting with key components of PRC2, namely enhancer of zeste homolog 2 (EZH2), and suppressor of zeste 12 homolog (SUZ12),^7^ CXorf67 has garnered significant attention. Numerous researchers have since become increasingly interested in the detailed molecular characterization of its functional domains.

The acquisition of DNA damage is an early driving event in tumorigenesis. Premalignant lesions often exhibit activation of the DNA damage response, while the inactivation of DNA damage checkpoints facilitates malignant transformation.^8^ For instance, in chronic myeloid leukemia, erroneous repair of DNA double-strand breaks can lead to chromosomal rearrangements, thereby promoting tumor development through the generation of oncogenic fusion proteins.^9^ However, DNA damage also represents a targetable vulnerability of cancer cells. Exogenous factors, such as ionizing radiation, ultraviolet light, and chemotherapeutic agents, can induce structural alterations in DNA through base modifications, bulky helix-distorting lesions, or DNA crosslinking, ultimately resulting in impaired repair and accumulation of cell death.^10^ Previous studies have suggested that the expression of CXorf67 is associated with the sensitivity of cancer cells to DNA-damaging agents, including camptothecin, etoposide, and doxorubicin.^11^ Therefore, CXorf67 is likely to play a role in the DNA damage response, thereby influencing tumor progression.

Given the current research on the function of CXorf67, it remains a recently characterized protein with largely undefined biological functions and is currently supported only as a germ cell-specific gene. To date, no evidence has implicated it in development, metabolism, or immune regulation. However, we have synthesized its biological significance, particularly emphasizing its potential role in epigenetics, as well as its function relevant to DNA damage in the development of diverse diseases. This could have profound implications for the involvement of CXorf67 in tumorigenesis and development.

## The structures and functions of CXorf67

**Figure 1:**
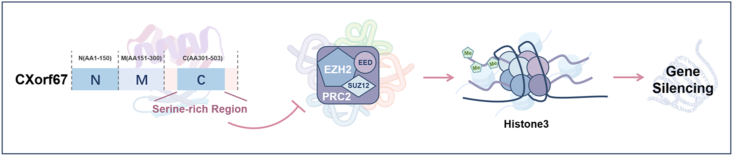
The schematic diagram of the CXorf67 protein structure. Through its C-terminal serine-rich domain, CXorf67 binds to and suppresses the enzymatic activity of EZH2 within the PRC2 complex, which is responsible for catalyzing H3K27 di- and tri-methylation and consequent gene silencing.

CXorf67 is predicted to be an intrinsically disordered protein, with only a serine-rich domain identified at its C-terminus.^12^ Researchers have hypothesized that CXorf67 may exert its function through the serine-rich domain located at its C-terminus, and it is indicated that the C-terminal region of CXorf67 is sufficient to bind and inhibit EZH2.^12^ This is attributed to the strong similarity between the 13-amino acid peptide sequence spanning amino acids 404 to 416 of CXorf67 and a mutant sequence of histone H3, a known downstream effector of EZH2 (Fig. 1). Additionally, arginine 405 (R405) is required for the interaction with EZH2.^13^

Mutations in histone H3 are typically associated with the suppression of EZH2 function.^12^ As previously mentioned, EZH2 is a key component of the PRC2, functioning primarily as a methyltransferase. It plays a critical role in the epigenetic maintenance of chromatin silencing through the tri-methylation of lysine 27 on histone H3 protein (H3K27me3) repressive mark.^14^ This mimicry enables CXorf67 to act as an endogenous inhibitor of PRC2 catalytic activity, thereby justifying its designation as EZHIP.

Acetylation (ac) and methylation (me) of histone lysine (K) residues play distinct roles in the regulation of most gene expression.^15^ While acetylation is generally associated with active transcription, lysine methylation can either activate or repress gene expression, depending on the specific target residue and the degree of methylation.^16^ PRC2 catalyzes the di- and tri-methylation of histone H3 at lysine 27 (H3K27me2/3), a repressive histone mark. The mechanism through which H3K27me2/3 exerts its function is likely the inhibition of transcriptional elongation, resulting in gene silencing.^17^ According to the published articles, when H3K27me3 modifications are deposited at promoter or enhancer regions of a gene, they can induce a transition of chromatin from an “open” euchromatin state to a “closed” heterochromatin state. This compaction of chromatin restricts access to the transcriptional machinery, thereby repressing gene transcription.^18^

Tobias et al identified CXorf67 as a tissue-specific cofactor of PRC2, highlighting its functional role in suppressing H3K27me3 deposition during spermatogenesis and oogenesis.^19^ The mRNA expression of CXorf67 is particularly elevated in the ovary, with significantly higher levels also observed in the testis compared with other tissues. This expression pattern reflects its role as a rapidly evolving protein, predominantly expressed in primordial germ cells during development and persisting in adult gonads. Loss of CXorf67 results in increased H3K27me2/3 levels during spermatogenesis and oocyte maturation, leading to impaired germ cell functionality.^20^

In addition to serving as a tissue-specific cofactor of PRC2, CXorf67 has also been identified as a cancer-testis antigen, suggesting its potential role in oncogenesis.^13^ In healthy adults, the expression of cancer-testis antigens (CTAs) is restricted to male germ cells, while ectopic expression of these antigens has been observed in tumor cells across various human cancers. Due to their ability to elicit robust cancer-specific immune responses, CTAs represent promising targets for highly specific immunotherapeutic interventions.^21^, ^22^, ^23^, ^24^, ^25^ Although CXorf67 is not included among the currently established CTAs, previous studies using RNA sequencing have identified 90 CTAs, including CXorf67, thereby defining the CTA landscape of non-small cell lung cancer (NSCLC) at the transcriptomic level.^13^ This suggests that CXorf67 may also function as a CTA in cancers. However, the precise mechanisms by which it acts as a CTA, particularly in NSCLC, remain poorly understood. This highlights the need for further investigation to uncover its downstream pathways and molecular mechanisms.

**Figure 2:**
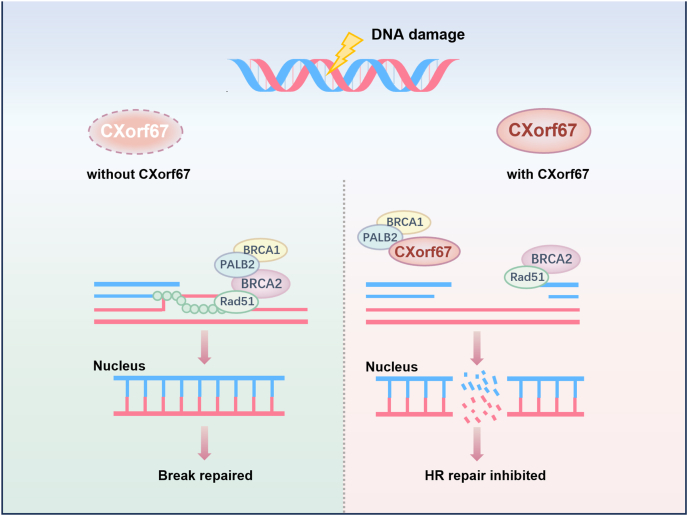
The involvement of CXorf67 in DNA repair. CXorf67 influences the function of PALB2 through its interaction with the BRCA1-PALB2-BRCA2 trimeric complex. When CXorf67 binds to PALB2, it compromises the assembly or activity of the complex, potentially impairing homologous recombination repair.

Recent studies, however, suggest that CXorf67 may have additional functions beyond its established role in the CXorf67-EZH2-H3K27 axis, as supported by existing literature and experiments. Partner and Localizer of BRCA2 (PALB2) encodes a recently identified protein that interacts with BRCA2, playing a role in its nuclear localization and stability. However, studies have shown that PALB2 primarily interacts with CXorf67 through its C-terminal WD40 domain.^26^ The role of CXorf67 in influencing PALB2 function arises from its interaction with the BRCA1-PALB2-BRCA2 trimeric complex.^27^ When CXorf67 binds to PALB2, the function of the BRCA1-PALB2-BRCA2 complex is consequently affected (Fig. 2).

When discussing the BRCA1-PALB2-BRCA2 axis, it is imperative to consider its crucial role in the DNA damage response. DNA double-strand breaks represent the most hazardous form of DNA damage. If left unrepaired or inaccurately repaired, they can result in cell death or genomic instability, ultimately leading to tumorigenesis.^28^ It is demonstrated that CXorf67 plays a pivotal role in human development by modulating chromatin states and influencing DNA transcription. Notably, its function appears to be closely associated with tumorigenesis, suggesting a potential role for CXorf67 in pathological conditions and highlighting its promising utilization.

## CXorf67-associated malignancies and its molecular mechanisms

### Posterior fossa a ependymomas

Ependymoma ranks as the third most common malignant tumor in the posterior fossa and represents a significant cause of neurological complications and mortality in children.^29^ Based on DNA methylation status and gene expression profiles, PF ependymomas can be classified into three subgroups: PFA, PFB, and PF-SE.^30^ Among the three subtypes, PFA is the most common and aggressive variant.^31^ The current standard of care involves surgical resection combined with radiotherapy. However, the difficulty in achieving complete tumor removal often results in poor clinical outcomes. CXorf67 is highly expressed in PFA ependymomas, where its promoter region has been found to be hypomethylated. In contrast, in normal tissues (except germline and placenta) and in all other PFA subtypes, the CpG islands within the gene promoter are typically highly methylated.^32^ This suggests that in PFA, promoter hypomethylation relieves transcriptional repression, thereby switching the gene from a silenced to a highly expressed state.^33^ In contrast, CXorf67 expression is absent in ependymomas of other molecular groups, suggesting a functional role for CXorf67 specifically within these tumors.^32^

Studies on the cell of origin in PFA have shown that CXorf67-positive PFA tumors encompass NSC-like, glial progenitor-like, and ependymoma-like cellular states.^34^ In contrast, CXorf67-negative PFAs are associated with a more favorable prognosis and are composed of more differentiated cell populations.^35^ As mentioned above, CXorf67 directly interacts with the catalytic site of the EZH2 subunit within the PRC2 complex through a highly conserved C-terminal peptide sequence. This sequence closely resembles the histone H3K27M mutant peptide, and its interaction effectively inhibits the methyltransferase activity of EZH2. Consequently, PRC2 function is disrupted, leading to a reduction in H3K27me3 levels. H3K27me3 plays a critical role in chromatin state regulation by facilitating chromatin compaction and suppressing transcriptional elongation, leading to gene silencing. Consequently, a reduction in H3K27me3 results in the derepression of PRC2 target genes, including those associated with neurodevelopment, which are up-regulated in PFA ependymomas with high CXorf67 expression.

Notably, previous studies have highlighted CDKN2A, a tumor suppressor gene, as being aberrantly silenced in PFA ependymomas with elevated CXorf67 expression.^36^ CDKN2A encodes key regulators of the G1-S cell cycle transition, which remain fully repressed in induced pluripotent stem cells (iPSCs) and other stem cell populations but are activated during cellular commitment.^37^ This suggests that in PFA ependymomas with high CXorf67 expression, although the overall levels of H3K27me3 are reduced, the remaining H3K27me3 accumulates in a localized manner at the CDKN2A locus, resulting in its aberrant silencing.

In addition to its role in the CXorf67-EZH2-H3K27 axis in PFA ependymoma pathogenesis, CXorf67 has also been implicated in DNA repair, suggesting a potential additional function in maintaining genomic integrity. It is well known that organisms have two major double-strand break repair pathways: non-homologous end-joining (NHEJ) and homologous recombination (HR). NHEJ is an error-prone end-to-end ligation process that can occur at any time in the cell cycle. By contrast, HR is an error-free process that occurs primarily in the S and G2 phases of the cell cycle.^38^

HR-mediated DNA repair in eukaryotic cells is a complex multistep process that requires the coordination of a set of proteins. Briefly, after γ-H2AX formation, BRCA1 recruits BRCA2 and RAD51 to the double-strand break site by interacting with PALB2. The BRCA1-PALB2-BRCA2 complex mediates the replacement of the replication protein A (RPA) complex by Rad51 and stimulates RAD51-dependent D-loop formation and strand invasion, thereby completing HR repair.^39^, ^40^, ^41^ Due to the amino acid sequence homology between the 13 residues near the C-terminal end of CXorf67 and the PALB2 binding motif of BRCA2, CXorf67 can bind to PALB2 and block the PALB2-BRCA2 interaction, and therefore, CXorf67 can competitively inhibit BRCA2 and thus inhibit HR-mediated double-strand break repair.^26^

It is demonstrated that PARP inhibitors exert their effects by binding to the catalytic sites of PARP1 or PARP2, preventing the proteins from dissociating from DNA damage sites. As a result, PARP remains bound to DNA, and during DNA replication, this binding leads to replication fork stalling, thereby impairing the progression of DNA replication.^42^^,^^43^ Consequently, PARP inhibitors induce synthetic lethality in tumors with defective homologous recombination (HR) repair. CXorf67 has been shown to inhibit HR repair in PFA ependymomas, and as a result, PARP inhibitors exert a potent cytotoxic effect in PFA ependymomas with high CXorf67 expression. This effect is further enhanced when PARP inhibitors are combined with radiotherapy, leading to increased tumor cell cytotoxicity.^26^

In summary, CXorf67 plays a pivotal role in PFA ependymomas by inhibiting PRC2 activity, modulating chromatin state and gene expression, and regulating DNA repair mechanisms. These findings provide a theoretical foundation for the development of novel therapeutic strategies targeting tumors with elevated CXorf67 expression.

### Diffuse midline glioma

Diffuse midline glioma (DMG) is a highly aggressive and destructive grade IV glioma, most commonly found in the brainstem, particularly in the pons (historically referred to as diffuse intrinsic pontine glioma, or diffuse intrinsic pontine gliomas).^44^ It is less frequently observed in the midbrain, thalamus, and spinal cord. Patients typically present with neurological symptoms, including cranial nerve deficits (*e.g.*, facial asymmetry and diplopia), cerebellar signs (*e.g.*, ataxia and dysarthria), and long tract signs (*e.g.*, hyperreflexia and weakness). The median overall survival for DMG patients is extremely short, typically ranging from 9 to 11 months, which makes it significant to investigate the multitude of complex biological sequelae that underpin tumor formation and disease progression.^45^

The WHO 5th edition of the Classification of Central Nervous System (CNS) Tumors designates DMG as “diffuse midline glioma, H3K27-altered”, a designation that includes the majority of diffuse intrinsic pontine gliomas and other tumors located along the midline (*e.g.*, brainstem, midbrain, thalamus, and spine).^46^ This classification recognizes molecular subtypes based on alterations to lysine 27 in histone H3 (H3 K27-altered),^47^ as well as cases featuring wild-type H3 alongside concurrent overexpression of the EZH2 inhibitory protein (EZHIP), also named as CXorf67. In the latest DMG classification, aberrant CXorf67 expression has expanded the spectrum of tumors with global H3K27me3 loss, leading to a redefinition of the subtype as H3K27-altered DMG, comprising both H3K27M and EZHIP-overexpressing variants.^48^ Therefore, the significance of CXorf67 in DMG is evident.

The role of CXorf67 in DMG cannot be discussed without addressing the H3K27 mutation present in these tumors. The oncogenic effect of the H3K27M mutation in DMG primarily occurs through the inhibition of PRC2, leading to widespread epigenetic alterations.^49^ H3K27M directly binds to the PRC2 complex, preventing its proper localization and function on chromatin, thereby hindering PRC2's ability to catalyze H3K27me3. Additionally, H3K27M impedes the binding of PRC2 to chromatin, further suppressing its role in gene regulation.^50^

As mentioned earlier, CXorf67 shares a similar amino acid sequence with H3K27M, suggesting that both CXORF67 and H3K27M act as structural and functional “mimics” of PRC2. By interacting with PRC2, they inhibit its activity, leading to a reduction in H3K27me3 levels, which may be similar to the function in PFA.

Although both H3K27M and CXorf67 inhibit PRC2 function, they do so through distinct mechanisms of action. CXorf67 interacts with the EZH2 subunit of PRC2 through its highly conserved serine region, thereby inhibiting EZH2's methyltransferase activity. In contrast, H3K27M exerts its effect not only by altering the local chromatin environment to recruit additional acetyltransferases to specific regions, but also by metabolically reprogramming glycolysis and the TCA cycle to produce more acetyl-CoA, ultimately increasing H3K27ac levels.^50^^,^^51^

H3K27ac and H3K27me3 are two distinct histone modifications, each playing a critical role in the activation and repression of gene expression, respectively. H3K27ac is typically associated with gene activation and cell proliferation, while H3K27me3 is linked to gene silencing and cell differentiation.^52^ Dysregulation of these modifications in tumors can lead to aberrant gene expression, thereby promoting tumorigenesis. In the case of DMG, H3K27M not only reduces the generation of H3K27me3 but also increases H3K27ac levels. This creates the potential for therapeutic strategies targeting the inhibition of histone deacetylases (HDACs), which have been shown to yield promising results in the treatment of H3K27M-DMG.^53^ A plausible molecular mechanism for enhancing the efficacy of HDAC inhibitors involves creating a widespread acetylation environment that induces the expression of endogenous retroviruses, thereby promoting immune responses,^54^ or activating pathways that enhance the regulation of cell differentiation, a process associated with H3K27M.^55^ However, H3K27-altered DMGs, in addition to the global loss of H3K27me3, are characterized by elevated levels of H3K27ac, H3K36me2, and aberrant DNA methylation. Although EZHIP-DMGs broadly resemble H3K27M-DMGs in their epigenetic landscape, they exhibit distinct molecular differences. Their DNA methylation profiles cluster more closely with H3K27M-DMGs than with EZHIP-PFAs, yet show increased hypermethylation at homeobox gene loci. Genetically, EZHIP-DMGs frequently harbor ACVR1 or EGFR mutations in a mutually exclusive manner, often accompanied by 1q gains, a pattern more reminiscent of H3.1K27M than H3.3K27M tumors.^51^ At the level of H3K27me3 retention and enhancer distribution, EZHIP-DMGs display both similarities and differences compared with H3K27M-DMGs and PFAs, suggesting divergent cells of origin.^56^

Collectively, these findings indicate that EZHIP-DMGs share core epigenetic features with H3K27M-DMGs while maintaining distinct genomic and epigenomic profiles, underscoring the need for comprehensive multi-omic and functional studies to clarify the role of CXorf67 in DMG pathogenesis.

### Osteosarcoma

Osteosarcoma (OS), the most prevalent primary malignant bone tumor of mesenchymal origin, is characterized by a bimodal age distribution, with incidence peaking both in adolescents during periods of rapid skeletal growth and in elderly individuals with preexisting bone disease.^57^ Despite advances in surgery and chemotherapy, the prognosis of OS remains unsatisfactory, particularly for patients with metastatic or recurrent disease, underscoring the urgent need to identify novel molecular targets. One major obstacle in OS treatment is its extensive genomic instability and chromosomal aberrations, which contribute to intra-tumoral heterogeneity, therapy resistance, and poor clinical outcomes. Conventional therapies, therefore, have substantial limitations, and the exploration of epigenetic drivers has emerged as a promising direction.^58^

Recent investigations have revealed aberrant expression of CXorf67 in approximately 20% of OS cases across two independent patient cohorts. Intriguingly, CXorf67 expression in OS cells shows a strong inverse correlation with the repressive histone mark H3K27me3, which is almost completely absent in EZHIP-positive tumors.^57^ Consistent findings from *in vivo* models further confirmed that genetic depletion of CXorf67 restores global H3K27me3 levels.^57^ These observations indicate that CXorf67 functions as an epigenetic regulator rather than merely a lineage marker. Mechanistically, CXorf67 interferes with Polycomb repressive complex 2 (PRC2) activity by inhibiting the conversion of H3K27me2 to H3K27me3.^59^ This suppression disrupts chromatin-mediated gene silencing, reshapes the global epigenetic landscape, and leads to the derepression of stem cell-associated transcriptional programs.^57^ As a result, CXorf67 promotes cellular plasticity and drives tumor initiation and progression through aberrant developmental reactivation.

The oncogenic consequences of CXorf67 in OS bear striking resemblance to those described in other tumor contexts. For instance, DMGs carrying the H3K27M mutation exhibit a profound loss of H3K27me3 and display marked sensitivity to EZH2 inhibition.^50^ In OS, pharmacological inhibition of EZH2 has likewise yielded encouraging preclinical evidence, showing that EZH1/2 orthosteric competitive inhibitors synergize with standard first-line chemotherapeutic agents.^57^ This suggests that targeting the PRC2 axis in OS may be therapeutically advantageous, particularly for patients harboring CXorf67 overexpression or EZHIP positivity. The parallel between OS and PFA ependymomas or DMGs highlights the generalizable mechanism of CXorf67 as a chromatin regulator, reinforcing its potential as a universal oncogenic driver across diverse cancer types.

Beyond its influence on histone methylation, CXorf67 exerts profound effects on the differentiation potential of mesenchymal stem cells, which are considered the putative cells of origin in OS. In functional assays, CXorf67 expression abrogates the ability of mesenchymal stem cells to undergo osteogenic, adipogenic, and chondrogenic differentiation, thereby disrupting their normal developmental trajectories. Instead, it aberrantly enhances their propensity to differentiate toward smooth muscle-like lineages, which not only compromises physiological bone formation but also fosters a tumor-permissive environment that accelerates disease progression.^57^ This shift in lineage specification provides an additional layer of evidence for the role of CXorf67 as a master regulator of epigenetic reprogramming in OS.

Taken together, these findings delineate a dual role of CXorf67 in OS pathogenesis: first, as an epigenetic modulator that reshapes the chromatin landscape to reactivate developmental gene programs, and second, as a determinant of lineage fate that restricts mesenchymal progenitor differentiation while redirecting cells toward aberrant smooth muscle pathways. From a translational perspective, CXorf67 not only contributes mechanistic insights into OS biology but also emerges as a clinically relevant biomarker. Its presence may predict responsiveness to EZH2 inhibitor–based therapeutic strategies, as residual H3K27me3 can still be targeted to confer therapeutic benefit in EZHIP-positive OS. Consequently, CXorf67 holds promise as both a pathogenic driver and a therapeutic guide, offering new avenues for improving the management of osteosarcoma.

### Endometrial stromal sarcoma

Malignant tumors of the female reproductive system, including cervical, ovarian, endometrial, and vulvar cancers, represent a significant global health burden.^60^ Among the more prevalent malignancies of the female reproductive system, such as ovarian cancer and endometrial cancer, numerous advanced treatment strategies have been developed by leading experts. Excluding carcinosarcomas, which are now classified as epithelial malignancies, endometrial stromal sarcoma (ESS) accounts for less than 1% of all uterine mesenchymal tumors and all uterine malignancies.^61^ Despite its low incidence, ESS is characterized by a high degree of malignancy, and its treatment options remain an area requiring further exploration and development.

In 2014, the WHO redefined the classification of ESS, a system that remains in use today. The term “endometrial stromal nodule” (ESN) was retained, while “endolymphatic stromal myosis” and “stromal sarcoma” were reclassified as “low-grade endometrial stromal sarcoma”, “high-grade endometrial stromal sarcomas”, and “undifferentiated endometrial stromal sarcoma”, respectively.^62^^,^^63^ High-grade ESS is typically defined as a poorly differentiated sarcoma, frequently characterized by active mitotic activity and pronounced nuclear pleomorphism.^64^

Gene fusions are frequently observed in endometrial stromal tumors, representing a structural chromosomal alteration in which DNA fragments from two genes are aberrantly joined through chromosomal rearrangement to form a fusion gene. The expression of such fusion genes can drive tumor initiation and progression. Among the most commonly described gene fusions in ESS is the t (7; 17) (p15; q21) chromosomal translocation.^65^ First identified in 1991 in a 58-year-old patient with metastatic, poorly differentiated endometrial stromal tumor, this translocation was subsequently characterized as the JAZF1/SUZ12 gene fusion, named after the involved genes.^66^

As research into endometrial stromal tumors deepens, an increasing number of gene fusions have been identified. Recent studies have demonstrated, using a combination of cytogenetic approaches and next-generation RNA sequencing, that MBTD1 and CXorf67 form a fusion gene involved in low-grade endometrial stromal tumors harboring the t (X; 17) (p11.2; q21.33) translocation.^67^

Similar to SUZ12, MBTD1 is a member of the Polycomb gene group (PcG), which is frequently involved in gene fusions associated with ESS.^68^ PcG proteins are critical epigenetic modifiers of chromatin, known to play pivotal roles in various biological processes, including development, stem cell self-renewal, X-chromosome inactivation, the differentiation of stem and somatic cells, and genomic maintenance, such as cellular responses to DNA damage.^69^

CXorf67 has been identified as an inhibitory protein targeting the EZH2 subunit of PRC2. Studies have demonstrated that CXorf67 specifically binds to EZH2, effectively suppressing PRC2 activity. Interestingly, while MBTD1 is not a subunit of PRC2, it is associated with PRC1, another major component of the PcG complexes. This observation raises the hypothesis that the MBTD1-CXorf67 gene fusion, identified in ESS, may influence PcG-mediated functions, potentially disrupting the interplay between PRC1 and PRC2.^67^

In general, the detection of fusion genes such as MBTD1-CXorf67 is emerging as a promising clinical tool for the diagnosis, staging, and classification of ESS. This is particularly relevant given that the differential diagnosis of uterine tumors largely depends on morphological evaluation of tissue specimens and immunohistochemical analysis. Notably, more than 60% of ESS cases are associated with chromosomal translocations involving PcG genes, including MBTD1, SUZ12, and PHF1. This raises the intriguing question of whether PcG inhibitors could serve as novel therapeutic candidates for ESS cases harboring PcG gene fusions. However, as ESS represents a rare subset of gynecological malignancies, the development of innovative treatment strategies remains an area requiring further investigation and dedicated research efforts.

### Non-small cell lung cancer

NSCLC is the most prevalent subtype of lung cancer, accounting for approximately 85% of all cases.^70^ Despite advances in early detection and conventional treatments such as surgery, chemotherapy, and radiotherapy, the prognosis for advanced-stage NSCLC remains poor. In recent years, immunotherapy has emerged as a transformative approach in the treatment of NSCLC, particularly with the advent of immune checkpoint inhibitors.^71^ Despite these advancements, challenges remain, including variable patient response, the development of resistance, and immune-related adverse events.^72^ As research progresses, understanding the molecular and immunological mechanisms underlying NSCLC will be critical to optimizing immunotherapeutic strategies and improving patient outcomes. Tumor-associated antigens are specific antigens that can be recognized by cellular or humoral immune responses. These include neoantigens generated by mutations, as well as endogenous proteins that are not expressed under normal physiological conditions. A prominent example of the latter category is CTAs.

As mentioned above, in NSCLC, CTAs are highly expressed, whereas their expression in normal tissues is primarily restricted to the testis. However, due to the absence of major histocompatibility complex (MHC) molecules in testicular cells, autoimmune responses are suppressed, preventing the effective presentation of these antigens to T cells.^73^^,^^74^ In contrast, in NSCLC, CTAs can serve as tumor-specific antigens, thus providing a rationale for the development of immunotherapeutic strategies targeting CTAs.

Utilizing RNA sequencing, immunohistochemistry, and mutation analysis, Hikmet et al have delineated the CTA landscape in NSCLC, revealing an association between elevated CXorf67 expression and increased local plasma cell infiltration.^75^ Although CXorf67 does not fall within the currently defined scope of CTAs, its chromosomal localization on the X chromosome and involvement in homologous recombination-mediated DNA repair pathways suggest that it could emerge as a potential target for immunotherapy in NSCLC, akin to other CTAs.

### Merkel cell carcinoma

Merkel cell carcinoma (MCC) is a rare yet highly aggressive cutaneous neoplasm with neuroendocrine features, first described in 1972 as “trabecular carcinoma of the skin”.^76^ The oncogenesis of MCC is associated with either clonal integration of Merkel cell polyomavirus (MCPyV, also known as human polyomavirus 5, or HPyV5) or chronic ultraviolet radiation exposure. Consequently, MCC is classified into two subtypes: virus-negative and virus-positive.^77^

Elevated expression of EZH2 has been observed in both virus-positive and virus-negative MCC cells, compared with normal skin cells and even other types of skin cancer.^78^ In MCC with overexpression of EZH2, two distinct patterns of H3K27me3 expression have been observed: cases exhibiting loss of H3K27me3 marking and others showing high levels of expression. Researchers have hypothesized that this discrepancy may be linked to the expression of CXorf67, which inhibits EZH2 activity. In virus-negative MCC cases, overexpression of CXorf67 may represent a mechanism underlying the loss of H3K27me3, distinct from the mechanism observed in virus-positive cases, where EZH2 expression is suppressed to reduce H3K27me3 levels. The study also highlights that overexpression of CXorf67 is found in certain squamous cell carcinoma cases of non-MCC skin tumors, in which H3K27me3 loss is similarly observed. This suggests that CXorf67 may play a role in the pathogenesis and progression of some skin tumors.

Overall, the role of CXorf67 in MCC appears to be primarily mediated through the EZH2-CXorf67-H3K27me3 axis. This mechanism aligns with our earlier hypothesis regarding the involvement of CXorf67 in cellular differentiation and cancer development, further supporting the potential of CXorf67 as a therapeutic target in cancer.

**Figure 3:**
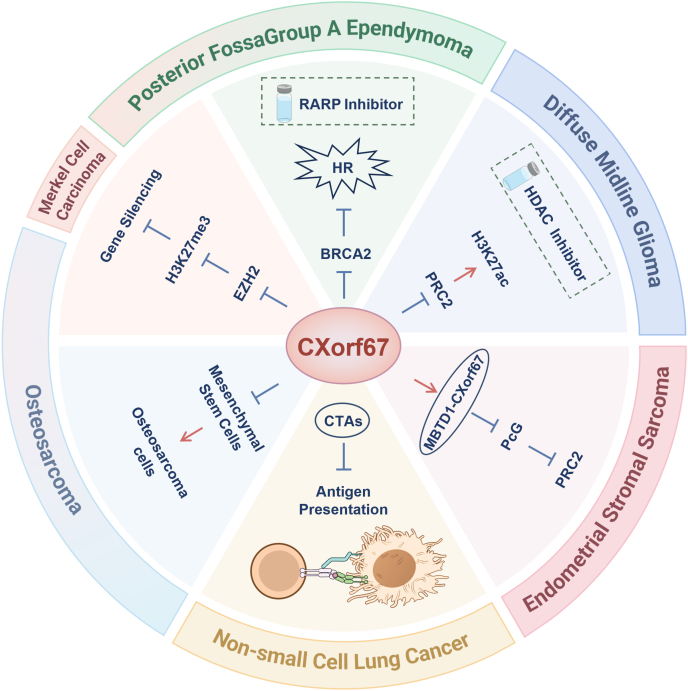
Panoramic figure integrating the molecular interaction network of CXorf67 with tumor lineage. The molecular mechanisms and potential roles of CXorf67 in the various tumors are illustrated in the figure.

All in all, in the cancers associated with CXorf67, except for small cell lung cancer, the pathogenesis is closely linked to the CXorf67-EZH2-H3K27me3 axis. This suggests a degree of universality in the role of CXorf67 in oncogenesis. However, whether CXorf67 exerts a similar function in other cancers remains unclear. Given its low expression in most tumors, it is uncertain whether CXorf67 acts as a pro-oncogenic factor in other malignancies. Consequently, its potential as a therapeutic target is still under investigation; these questions require further in-depth studies to elucidate the role of CXorf67 in cancer biology (Fig. 3).

## The potential application of CXorf67-related therapies in the treatment of cancers

Although CXorf67 has been identified as a potential therapeutic target in cancer, no specific treatments targeting CXorf67 have been developed to date. This underscores the urgent need to explore and establish effective therapeutic strategies for this target. Based on the potential downstream targets and functional roles of CXorf67, several candidate compounds with potential efficacy against CXorf67-associated pathways have been identified and are outlined below.

### PARP inhibitor

Poly (ADP-ribose) polymerase (PARP) inhibitors were initially developed as a targeted therapeutic approach for patients with BRCA-mutated cancers,^79^ leveraging the concept of synthetic lethality as their primary anti-cancer mechanism. In healthy cells, DNA damage response (DDR) pathways counteract the detrimental effects of DNA damage by detecting lesions, arresting the cell cycle, and facilitating DNA repair, thereby preserving genomic integrity.^80^ Central to DDR are the enzymes poly (ADP-ribose) polymerase 1 and 2 (PARP1 and PARP2), which function through the synthesis of PARylation as a post-translational modification.^81^ PARP1 plays a pivotal role in DNA repair by binding to damaged DNA at single-strand breaks (SSBs) and other lesions, mediating repair primarily via NHEJ. In addition to NHEJ, another, more precise DNA repair mechanism exists, known as homologous recombination (HR). HR accurately restores the original structure of damaged DNA molecules by using a homologous template, making it the default mechanism for repairing replication forks.^82^ This high-fidelity process is essential for maintaining genomic stability and preventing the accumulation of mutations.^83^

Early studies demonstrated that cells lacking BRCA are unable to mediate HR repair and are instead reliant on the NHEJ pathway for DNA damage repair. Compared with HR, NHEJ is a more error-prone process, often resulting in nucleotide deletions, insertions, and chromosomal translocations.^84^ This propensity for genomic instability is a key reason why BRCA deficiency significantly increases the risk of cancer development.

However, when PARP inhibitors are applied to cancer cells with BRCA deficiencies, the process of PARylation is inhibited, preventing the repair of SSBs via NHEJ. In the absence of functional BRCA, HR repair is also impaired. This dual inability to resolve DNA damage ultimately results in the accumulation of irreparable DNA lesions, leading to cell death. This phenomenon, where the deficiency of either gene alone has minimal impact on cells or organisms, but the simultaneous loss of both genes results in cell death, is termed synthetic lethality.^85^ Therefore, the cancer cell death induced by PARP inhibitors is a classic example of synthetic lethality.

Although the mechanism by which PARP inhibitors exert cytotoxicity in CXorf67-high PFA tumors remains elusive, the pronounced suppression of tumor growth upon PARP inhibitor–radiotherapy co-treatment indicates a strong mechanistic link to DNA damage repair. Plus, studies suggest that PARP plays a role in transcriptional regulation through histone modification and the PARylation of other transcription factors.^86^ Additionally, PARylation has been shown to induce the inactivation of EZH2,^87^ which may contribute to the tumoricidal effects observed in CXorf67-expressing PFA tumors.

### EZH2 inhibitor

As mentioned above, the catalysis of H3K27me3 is a key epigenetic modification involved in gene expression regulation. This specific mark is associated with transcriptional repression and is catalyzed by PRC2, which includes the EZH2 subunit.^88^ The H3K27me3 modification plays a crucial role in maintaining cellular identity and regulating processes such as development, differentiation, and silencing of developmental genes.

Experimental evidence has demonstrated that EZH2 inhibitors exert significant inhibitory effects on diffuse midline gliomas harboring the H3K27M mutation.^89^ These inhibitors function by directly binding to EZH2, thereby suppressing its catalytic activity and preventing PRC2-mediated trimethylation of H3K27.^89^ Further studies have shown that H3K27me3 acts to repress the expression of the tumor suppressor gene p16INK4A.^89^ Upon treatment with EZH2 inhibitors, the increased expression of p16INK4A leads to its binding with CDK4/6, thereby inhibiting their activity.^89^ This, in turn, halts cell cycle progression by blocking the G1/S transition, preventing DNA synthesis, and ultimately inhibiting cellular proliferation.

Although there are no clinical studies directly investigating the use of EZH2 inhibitors in PFA tumors, research has indicated that in PFA tumors with high CXorf67 expression, CXorf67 inhibits PRC2 activity through a mechanism similar to H3K27M.^36^ Compared with choroid plexus tumors with low CXorf67 levels, PFA tumors with high CXorf67 expression exhibit reduced CDKN2A expression, while the methylation activity of PRC2 at CDKN2A loci is preserved. This suggests that the inhibitory effect of EZH2i on methylation could potentially have a similar suppressive effect on PFA tumors with high CXorf67 expression.

In summary, although direct clinical evidence is lacking, the parallels between H3K27M-driven gliomas and CXorf67-high PFA tumors strongly suggest that EZH2 inhibition could effectively counteract CXorf67-mediated suppression of PRC2 activity. By restoring proper regulation of CDKN2A and cell cycle control, EZH2 inhibitors may represent a promising therapeutic strategy for suppressing tumor growth in PFA with elevated CXorf67 expression. Moreover, given the mechanistic relevance of CXorf67 as a tumor-associated target, it is of considerable interest to explore whether the therapeutic potential of EZH2 inhibition could extend to other malignancies characterized by high CXorf67 expression.

### HDAC inhibitor

After undergoing various post-translational modifications, histones regulate chromatin structure and gene expression.^90^ Alterations in histone acetylation are often associated with the development of cancer.^91^ As previously mentioned, a dynamic balance exists between H3K27me3 and H3K27ac in the cellular context; disruption of this equilibrium usually leads to the onset and progression of cancer. For instance, in DMG harboring the H3K27M, the mutated H3K27 locus impairs the methyltransferase activity of PRC2, resulting in a decrease in overall H3K27me3 levels and an accompanying increase in H3K27ac levels.^44^

Beyond DMG, HDACs are also closely implicated in the pathogenesis of various other cancers. Elevated expression of HDAC1/2/3 has been associated with poor prognosis in gastric and ovarian cancers,^92^^,^^93^ while increased HDAC8 expression correlates with advanced stages and reduced survival rates in neuroblastoma.^94^^,^^95^ Studies have also demonstrated that HDAC inhibitors can suppress cancer proliferation by down-regulating cyclin-dependent kinases CDK4/6,^91^ a mechanism reminiscent of the antiproliferative effects exerted by EZH2i.

In DMG with high CXorf67 expression, the structural similarity to H3K27M results in a global reduction of H3K27me3 levels. Given the dynamic balance between H3K27me3 and H3K27ac, treatment with HDAC inhibitors has been observed to partially reduce H3K27ac, thereby exerting some degree of tumor-suppressive effects. However, the clinical application of HDAC inhibitors, such as panobinostat, remains challenging due to limited therapeutic efficacy, severe toxicity, and poor blood–brain barrier (BBB) permeability.^96^

Collectively, these findings highlight the critical interplay between histone acetylation and methylation in tumorigenesis. While current HDAC inhibitor therapies face limitations in clinical translation, the mechanistic overlap between CXorf67-mediated disruption of H3K27me3 and the consequent compensatory gain of H3K27ac underscores the therapeutic relevance of targeting HDACs. Thus, HDACs, together with CXorf67, represent promising epigenetic vulnerabilities that warrant further exploration as potential therapeutic targets across cancers characterized by CXorf67 overexpression.

## Conclusion

Cxorf67, encoded by a gene located on the X chromosome, is an understudied protein with emerging roles in epigenetic regulation and tumorigenesis. It interacts with key components of the PRC2, specifically EZH2 and SUZ12, to inhibit their activities, thereby affecting histone modifications such as H3K27me3. This interaction is mediated through a conserved C-terminal domain in Cxorf67.

Owing to its high expression in PFA and the shared structural similarity to H3K27M in DMG, as well as its identification as a potential CTA, the mechanisms through which CXorf67 contributes to cancer remain largely enigmatic and warrant further investigation. However, CXorf67 exhibits low expression in most cancers and normal human tissues, suggesting its potential role as a tumor suppressor. Previous studies have indicated that CXorf67 may regulate tumor initiation and progression through epigenetic mechanisms by modulating DNA silencing. Beyond brain tumors, such as PFA, research on CXorf67 as a therapeutic target and oncogenic factor in other cancers remains scarce. This paucity of studies can be attributed to its relatively recent discovery, the rarity of its occurrence, and the lack of adequate preclinical models.

Even within PFA and DMG, the functional roles of CXorf67 remain incompletely elucidated. For instance, it is associated with the sensitivity of cancer cells to DNA-damaging agents, which are commonly used in radiotherapy and chemotherapy. CXorf67 may influence the efficacy of these treatments by modulating the DNA damage response. As such, CXorf67 holds significant potential as a prospective therapeutic target in oncology, warranting further in-depth investigation. The previously discussed CXorf67-related therapeutics largely center on its interaction with EZH2 and its role in inhibiting histone methylation. This focus suggests that additional mechanisms of CXorf67 remain unexplored. Particularly as a CTA and given its involvement in DNA damage response, CXorf67 presents an intriguing avenue for further investigation and discovery by the scientific community, potentially leading to novel therapeutic strategies that could enhance the effectiveness of radiotherapy and chemotherapy in cancer treatment.

## CRediT authorship contribution statement

**Xinning Yu:** Writing – original draft, Visualization, Resources, Investigation, Data curation. **Huatao Wu:** Writing – original draft, Visualization, Resources, Investigation, Data curation. **Yangzheng Lan:** Visualization, Investigation, Data curation. **Wenjia Chen:** Investigation, Data curation. **Jing Liu:** Writing – review & editing, Visualization, Supervision, Resources, Project administration, Investigation, Funding acquisition, Data curation, Conceptualization.

## Funding

This work was supported by the 10.13039/501100001809National Natural Science Foundation of China (No. 82273457), the 10.13039/501100021171Guangdong Basic and Applied Basic Research Foundation (China) (No. 2023A1515012762), Science and Technology Special Project of Guangdong Province, China (No. 210715216902829), and “Dengfeng Project” for the construction of high-level hospitals in Guangdong Province (China)—the First Affiliated Hospital of Shantou University Medical College Supporting Funding (No. 202003-10).

## Conflict of interests

The authors declared no conflict of interests.
